# Smart city and earnings management: Evidence from China

**DOI:** 10.1371/journal.pone.0301025

**Published:** 2024-04-02

**Authors:** Dan Sun, Chiping Chen

**Affiliations:** 1 School of Economics, Hunan Institute of Engineering, Xiangtan, Hunan Province, China; 2 Department of Economics and Management, Changsha University, Changsha, Hunan Province, China; Shanghai Business School, CHINA

## Abstract

Smart cities improve services for businesses, among many other benefits. A comprehensive understanding and effective utilization of these advantages is crucial for promoting business development. Using panel data from Chinese listed companies (2010–2020), this study employs a multi-stage DiD model to investigate the impact of smart cities on corporate earnings management. The findings indicate that the smart city pilot policy has significantly reduced corporate earnings management. Further analysis suggests that smart cities primarily reduce earnings management by improving firms’ external information environments. Additionally, the results show that the policy impact of smart cities is more significant in regions with lower regulatory intensity or higher marketization levels, compared to regions with higher regulatory intensity or lower marketization levels. Similarly, firms in less concentrated markets or those more closely related to smart city development tend to experience greater reductions in earnings management due to smart city construction, unlike firms in more concentrated markets or those less involved. Finally, this paper offers several brief suggestions.

## 1 Introduction

Rapid urbanization and significant demographic shifts have aggravated the problems of environmental pollution, resource shortage, and traffic congestion [1,2], thereby placing enormous pressure on urban governance [3]. The smart city concept is considered the most attractive solution to such challenges and is continuously explored by various countries. For example, in the United Kingdom, one-third of cities with populations exceeding 100,000 exhibit clear smart city ambition and have launched substantial related initiatives [4,5]; in the United States, two-thirds of cities have invested in smart technology [6]; in Europe, both national governments and the European Union are making significant investments in smart city projects [7]. With the growing practical knowledge in smart city development, scholars are expanding their focus beyond the contribution of these advancements to urban sustainability, delving into the broader economic implications that smart cities present. Similarly, this paper focuses on analyzing the impact of smart city construction on earnings management from a corporate perspective.

Smart cities integrate various intelligent sensors into urban infrastructure, thereby forming an Internet of Things (IoT) through signal interconnection. Correspondingly, the IoT detects, collects, assesses, and integrates the big data generated from urban operations, in order to improve resource allocation efficiency and ensure smart governance, smooth operation, and sustainable development of the city [8–10]. The extant literature on the impact of smart cities on firms primarily focuses on the information effects of these developments.

From the perspective of information effects, smart cities significantly enhance the corporate information environment. The development of smart city improves the interconnection, interoperability, and interaction among various urban elements, thus enabling efficient processing and swift dissemination of substantial information [11,12]. The swift flow of data consistently broadens the scope of accessible information while reducing search costs and revealing more profound layers of data [13]. Consequently, firms emerge as major beneficiaries of information technology integration, enabling them to minimize the waste of non-essential resources and enhance resource allocation efficiency, thereby improving their total factor productivity [14]. Concurrently, smart cities leverage diverse digital technologies to facilitate information exchange among individuals and organizations, effectively reducing the costs of information exploration and promoting innovation [13]. Moreover, the advantages of optimized information management extend to the stock market. Smart cities serve as an optimal platform for the prompt dissemination of enterprise information, reducing information asymmetry between enterprises and stakeholders. This helps mitigate adverse selection and herd behavior in the investment process, thereby improving the efficiency of the capital market [15].

Though there is a consistent interest from different disciplines, studies on the economic impacts of smart cities on firms demonstrate certain limitations. First, while extant literature has extensively studied the impact of smart city construction on firms, there is limited research on the link between smart city development and firms’ information disclosure strategies. Intuitively, smart cities can facilitate the convenience, accuracy, and timeliness of information transmission, potentially influencing firms’ information disclosure strategies, notably in terms of earnings management. Second, most studies related to this topic face challenges in effectively addressing endogeneity issues due to omitted variables, making it difficult to ascertain robust causal relationships between the study variables.

In order to bridge the research gap discussed above, this study utilizes panel data from Chinese listed companies and aims to explore the impact of smart city construction on corporate earnings management. This research paper contributes to the existing literature in the following ways. First, to the best of our knowledge, this is the only study that investigates the impact of smart city construction on corporate earnings management. Therefore, this shall not only enrich the present literature on the economic influences of smart cities but also serve as a significant supplement to the studies on the factors affecting corporate earnings management. Secondly, our research indicates that smart city development effectively enhances the external information environment of corporations. This not only represents an underlying mechanism by which smart city construction impacts corporate earnings management, but it also serves as a potential channel influencing various other aspects of business operations, thus providing significant insights for future research. Lastly, this research not only examines the influence of smart city development on corporate earnings management but also identifies potential individual heterogeneity in this effect, thereby offering richer insights into the effects of smart city construction on corporate earnings management.

## 2 Research background and research hypotheses

### 2.1 Smart cities pilot project in China

While sharing commonalities in utilizing technology to enhance public services and economic growth, Chinese cities have adopted approaches to smart city construction that differ from those in developed countries [16]. Chinese smart cities exhibit a top-down construction approach [17]. Benefiting from significant policy and financial support from the central government, local governments play a pivotal role in developing the smart technology industry, protecting the environment, and building intelligent infrastructure [18].

Despite a later start compared to developed countries, the construction of smart city in China has made rapid progress, driven by strong policy support [19]. In 2012, the Ministry of Housing and Urban-Rural Development of China issued the “Interim Measures for the Administration of the National Smart City Pilot” and subsequently initiated the pilot program for smart cities in the first batch of 90 areas [20]. According to the "National Smart City (District, Town) Pilot Index System," pilot cities are mandated to develop smart cities focusing on four key indicators: support system and infrastructure, intelligent construction and livability, smart industry and economy, and smart management and services [21]. Additionally, the construction of smart cities is guided by eleven secondary indicators, such as network infrastructure, databases, and public platforms. By 2014, a total of 290 cities or regions in China had sequentially launched smart city pilot projects [22].

As of 2019, the cumulative number of smart city pilot projects identified in various sectoral reports and development strategies in China exceeded 700 [23]. Regarding the quantity of smart cities constructed and the scale of their development, China has advanced to a leading position globally [2]. Cutting-edge technologies, such as big data, the IoT, cloud computing, and artificial intelligence (AI), are extensively integrated into the framework of smart city construction [20].

### 2.2 Research hypotheses

The principal-agent problem presents a significant factor in corporate earnings management [24]. In an agency relationship where both parties aim to maximize their utility, the agent might not always act in the principal’s best interests [25]. Consequently, the principal-agent problem arises if there is a conflict of goals between the two parties and if monitoring the agent’s actions becomes challenging or expensive for the principal [26]. Under the principal-agent association, managers perceive the reported earnings as a key indicator for stakeholders to evaluate a firm’s financial performance, executive remuneration, and future survival prospects [27]. As a result, agents who aim to maximize their personal gain may intend to control or adjust business performance through accounting means when the firm’s operating performance falls short of expectations, leading to earnings management [28–30].

In the principal-agent relationship, management is encouraged to engage in earnings management when presenting financial statements. To limit this opportunistic behavior and oversee contract issues between management and investors, adopting a more rational ownership structure and optimizing the board structure are considered crucial approaches [24]. Research indicates that increased controlling shareholder voting rights correlate with reduced earnings management, and an inverse U-shaped relationship exists between insider ownership and earnings manipulation [31]. Expanding the board of commissioners and increasing independent commissioners can reduce earnings management, while the CEO’s dual role as chairperson often has a positive impact on earnings management [32]. Younger audit committee financial experts (ACFEs) tend to exercise more efficient control over firm management, thus better mitigating earnings management compared to their older counterparts [33]. Increasing the number of independent directors on the audit committee strengthens its oversight of earnings quality, leading to a reduction in corporate earnings management [34]. Female CEOs, known for their risk-averse nature, are generally more cautious in engaging in earnings manipulation, particularly during bearish market periods [35]. The aforementioned ownership structures and board characteristics more efficiently oversee managerial activities, thus constraining their ability to engage in earnings management [31].

Another significant factor in enabling earnings management is the information asymmetry between the management (agent) and the owner (principal) [24]. High information asymmetry leads to stakeholders lacking the resources, incentives, and access to relevant information necessary for effective oversight of managerial actions, thus contributing to the practice of earnings management [36,37]. In this context, addressing information asymmetry is crucial in curbing corporate earnings management. In modern corporations, owners can mitigate information asymmetries by overseeing agent actions and gaining access to their firm’s internal information flows [38]. Research indicates that disclosure of third-party online sales reduces earnings management by decreasing its benefits and increasing the risk of its detection [39]. Implementing the standardized eXtensible Business Reporting Language (XBRL), a digital format known for its efficiency and flexibility that offers a universal framework for global financial communication, markedly reduces corporate earnings management in financial reports [40]. Common institutional ownership substantially lowers the costs of information acquisition and processing for institutions, thereby enhancing monitoring efficiency and reducing earnings management [41].

Expanding on existing research, this study investigates whether smart city construction can impact corporate earnings management by addressing information asymmetry. A primary goal of smart city construction is the integration of pertinent urban big data (e.g., business data, city infrastructure IoT big data, and government service data) to facilitate efficient predictive analysis and informed decision-making by city managers, firms, and individuals [42]. Utilizing modern information and communication technologies, smart cities are capable of comprehensively and transparently collecting information, securely and broadly disseminating information, and intelligently and efficiently processing information [43]. This framework supports synchronized corporate information disclosure and utilization through big data technologies, significantly enhancing the efficiency of information production and dissemination, thereby reducing information asymmetry [44,45]. In a more transparent information environment, corporate management is expected to have reduced discretion in asset valuation and profit recognition; while external stakeholders can more easily and cost-effectively capture private information that management may attempt to hide [41,46]. As a result, this facilitates timely identification of accounting manipulation behaviors, increases the challenges in corporate earnings management, and helps curb such malpractices [39].

In China, smart city construction emphasizes comprehensive data development in government affairs, management, and services, with the objective of facilitating data co-creation and sharing, reducing information asymmetry in the market [10,16]. Such measures are likely to mitigate earnings management and enhance the quality of financial information [44]. Building on this foundation, the following hypotheses are proposed in this study:

H1: Smart city construction curbs corporate earnings management and improves the quality of accounting information.
H2: Smart city curbs the behaviors of corporate earnings management by improving the external information environment.

## 3 Research methodology

### 3.1 Data source and processing

Primarily, firm-specific micro-level data are sourced from the China Stock Market and Accounting Research (CSMAR) database, while data related to smart city pilot projects are derived from the list of pilot cities issued by the Ministry of Housing and Urban-Rural Development of China. Additionally, city-level data are obtained from the annual "China City Statistical Yearbook" and statistical yearbooks/gazettes of provincially-administered counties and county-level cities, autonomous prefectures, and leagues. Meanwhile, linear interpolation is employed to address missing values in city-level data.

The following samples are excluded from this research study: first, those from the financial industry, due to their significant differences from other industries; second, ST, *ST companies, along with those that are delisted or suspended; third, any observations with missing variables or exhibiting abnormal financial status, such as financial leverage exceeding 100%. Additionally, the sample period, spanning from 2010 to 2020, was selected to exclude any atypical effects on corporate behavior due to the Global Financial Crisis and the COVID-19 Pandemic, thereby maintaining the robustness of the research findings. Given that the smart city pilot projects were implemented between 2012 and 2014, this study includes samples from up to four years prior to and eight years following the policy implementation, a timeframe considered sufficient to ensure the study’s robustness. Furthermore, referring to previous studies [47,48], all continuous firm-level variables are trimmed at the 1% level on both tails to minimize outlier effects. After excluding these samples, 21,134 observations remain.

### 3.2 Model specification

Since smart city construction is assumed to be an exogenous shock, this research incorporates a multi-period difference-in-differences (DID) model to predict the relationship between smart city pilot projects and corporate earnings management, as demonstrated by Eq (1).

$$Y_{ijct}=\beta_{0}+\beta_{1}∙{Smart\;City}_{ct}∙{post}_{t}+\beta_{2}∙{Smart\;City}_{c}+\beta_{3}∙{post}_{t}+\beta_{2}X_{ijct}^{\prime }+u_{j}+u_{c}+u_{t}+\varepsilon_{ijct}$$ (1)

Where Y_ijct_ represents the variable measuring the degree of earnings management by firms; subscripts i, j, c, and t denote firms, industries, cities, and years, respectively. Smart City_ct_ refers to the pilot smart cities (treatment group), and post_t_ is a time dummy variable, with 1 indicating the post-treatment period and 0 otherwise. Moreover, X’_ijct_ represents a series of control variables that influence the degree of earnings management by firms. u_j_, u_c_, and u_t_ represent industry fixed effects, city fixed effects, and year fixed effects, respectively, controlling for industry and city characteristics that do not change over time, as well as macroeconomic shocks at the national level. As the fixed effects for cities and years are controlled, the dummy variables Smart City_ct_ and post_t_ are absorbed, implying that their coefficients (β_2_ and β_3_) will not appear in the regression results. Lastly, ε_ijct_ represents the random error term.

#### 3.2.1 Dependent/Explained variable: Accrual-based earnings management (AEM)

The stable development of the capital market is subject to high-quality accounting information. As an imperative component of accounting information, earnings information reflects the financial status and operational outcomes of firms over a specific period. Additionally, it serves as an important means of information for stakeholders to assess and make decisions about firms [27]. The modified Jones model [49] is adopted in this paper to calculate the accrual-based earnings management (AEM) for each firm.

$$\frac{{TA}_{i,t}}{A_{i,t-1}}=\beta_{0}\frac{1}{A_{i,t-1}}+\beta_{1}\frac{\Delta {REV}_{i,t}}{A_{i,t-1}}+\beta_{2}\frac{{PPE}_{i,t}}{A_{i,t-1}}+\varepsilon_{i,t}$$ (2)

$${NDA}_{i,t}=\hat{\beta_{0}}\frac{1}{A_{i,t-1}}+\hat{\beta_{1}}\frac{\Delta {REV}_{i,t}}{A_{i,t-1}}+\hat{\beta_{2}}\frac{{PPE}_{i,t}}{A_{i,t-1}}$$ (3)

$${DA}_{i,t}=\frac{{TA}_{i,t}}{A_{i,t-1}}-{NDA}_{i,t}$$ (4)

Where TA_i,t_ represents total accruals in year t for firm i; A_i,t-1_ indicates the total assets at the end of the previous year, used to eliminate the scale effect; ΔREV_i,t_ and ΔREC_i,t_ signifie the change in operating revenue and accounts receivable, respectively, for the current year. PPE_i,t_ refers to net fixed assets in the current year, while NDA_i,t_ and DA_i,t_ represent non-discretionary accruals and discretionary accruals, respectively. The estimation process is as follows: firstly, estimate Eq (2) on a yearly and industry basis; secondly, use the estimated coefficients in Eq (3); and finally, calculate the discretionary accruals (DA) as per Eq (4), where the absolute value of DA represents the accrual earnings management (AEM). In this study, AEM serves as a proxy for earnings management. Apparently, a higher value of AEM indicates a higher degree of earnings management, while a lower value signifies a lower degree of earnings management.

#### 3.2.2 Core explanatory/independent variable: Pilot smart city

Between 2012 and 2014, the Ministry of Housing and Urban-Rural Development, along with the Ministry of Science and Technology, approved three batches of smart city pilot projects. Given that some pilot zones are at the county, town, or district level, classifying all cities containing those regions as smart cities may underestimate the policy impacts of smart city pilots. Consequently, this study categorizes smart cities in two ways: firstly, in the light of the present literature [14,50], a city is considered a smart city only if all its regions fall within the smart city pilot scope (denoted as Smart City). In this case, Smart City_ct_ is assigned a value of 1; otherwise, it is assigned 0. Secondly, a city is categorized as a smart city if some of its regions (at the county level or above) are part of the smart city pilot scope, with the population ratio of the pilot area to the entire city serving as a proxy for smart city (denoted as Smart City*). For example, since Shangcheng District of Hangzhou became a smart city pilot region in 2012, Smart City*_Hangzhou,2012_ = population of Shangcheng District/population of Hangzhou. This continuous variable, Smart City* identifies a city’s involvement in smart city pilot projects by measuring the percentage of its population residing in these pilot areas. Moreover, population data for these areas are sourced from the seventh national census of China.

#### 3.2.3 Control variables

In alignment with Chen et al. [51] and Jiang et al. [52], this study incorporates firm-specific variables known to be correlated with earnings management. Initially, the study controls for firm size (Size) and age (ListAge), as prior research suggests that larger and older companies tend to engage less in earnings management [53,54]. Previous studies, such as Kim et al. [40], indicate that firms with operating losses are more likely to engage in earnings manipulation. Therefore, we include loss (Loss), assigned a value of one for firms experiencing operating losses. The study includes firm leverage (Leverage), defined as the ratio of total liabilities to total assets, given that firms with high leverage may be more incentivized to manage earnings to prevent breaches of debt covenants [40]. Additionally, to account for the potential correlation between accruals and cash flows as suggested by Kothari et al. [55], cash flow from operations (CFO) is included in our model. To address the potential correlation between sales growth and earnings management, as identified by Collins et al. [56], we include sales growth (Growth) as a control variable.

Furthermore, in line with findings from Guo and Ma [57], which suggest that earnings management is influenced by various ownership motivations, this study included several variables representing different types of firm ownership. State-owned enterprises (SOE), in which the government is the actual controller, are included based on Cheng et al.’s [58] finding that such enterprises are less inclined to engage in earnings manipulation. The shareholding percentages of the largest (Top1) and the top 10 shareholders (CR10) are included to control for the potential influence of ownership concentration on earnings management [52,57]. Similarly, common institutional ownership (CIO), defined as the total ownership percentage held by common institutional investors, is controlled for, as it can mitigate earnings management [41].

Furthermore, prior studies have demonstrated that the characteristics of the board of directors significantly influence a firm’s accounting accruals [59,60]. Consequently, the study controls for board size (BSize), board independence (BInd), and the number of board meetings (Meetings). Notably, previous studies have yielded varied results on the relationship between CEO duality and earnings management [60]. However, considering the diminished monitoring effectiveness of a board when the CEO also serves as the chairperson, we include CEO duality (Dual) as a control variable. Additionally, previous research suggests a correlation between CEO compensation levels and earnings management [61], prompting the inclusion of executive compensation (ExePay) as a control variable. Moreover, the existing literature, such as Krishnan [62], indicates that Big 4 auditors are more effective at constraining earnings management. As a result, an indicator variable, Big4, is included, assigned a value of one for firms audited by any of the international Big Four auditors, and zero otherwise. Similarly, past research has established a positive association between modified audit opinions (MAOs) and earnings management [63]. Therefore, we control for Audit Opinion (Opin), signifying an unqualified audit opinion.

Lastly, to account for the potential influence of regional economic conditions on earnings management, as highlighted by Jiang et al. [52], this study incorporates several variables: city-level GDP per capita (pcGDP); the share of secondary industry in local GDP (SIR), reflecting industrial structure; and city-level GDP growth rate (GDPgrowth). Additionally, in line with Chen’s [14] research, we consider city population size (Pop) as a measure of city size. Detailed definitions and measurements of all variables are outlined in Table 1.

**Table 1:** Definition of main variables.

Variable Category	Variable Name	Variable Symbol	Variable Description
Dependent Variable	Accrual Earnings Management	AEM	The absolute value of discretionary accruals serves as a proxy of earnings management.
Independent Variable	Smart City Pilot	Smart City	A city is classified as a smart city and assigned a value of 1 if all its regions are within the smart city pilot scope; otherwise, it is assigned a value of 0.
		Smart City*	A city is considered a smart city if any of its regions are part of the smart city pilot, using the population ratio of the pilot area to the entire city as an indicator.
Firm-level Control Variables	Firm Size	Size	Logarithm of the total assets at the end of the year for firms.
	Firm Age	ListAge	Logarithm of the number of years since the company went public plus 1.
	Leverage ratio	Lev	Total liabilities divided by total assets.
	Cash Flow Return on Assets	CFO	The total operating cash flow scaled by the total assets.
	Sales Growth Rate	Growth	The increase of annual revenue divided by previous year’s revenue.
	State-owned enterprises	SOE	A dummy variable that equals to one if a firm is a state-owned enterprise.
	Loss	Loss	A value of 1 is assigned when the net profit is negative, and 0 otherwise.
	Ownership Concentration	Top1	The shareholding percentage of the largest shareholder.
		CIO	The total shareholding of common institutional investors.
		CR10	The total shareholding of top 10 shareholders.
	Board Size	BSize	The natural logarithm of the number of members on the board of directors.
	Board independence	BInd	The ratio of independent directors to the total number of directors in firms.
	Board meetings	Meetings	The natural logarithm of the number of board meetings.
	Executive Compensation	ExePay	The logarithm of the total compensation for the top three executives.
	CEO Duality	Dual	Assign a value of 1 in case the chairman and CEO positions are held by the same individual; otherwise, assign a value of 0.
	Big Four Auditors	Big4	The presence of auditors from the Big Four Accounting Firms.
	Audit Opinion	Opin	A value of 1 is assigned for an unqualified audit opinion, and 0 otherwise.
City-level Control Variables	Per Capita Regional GDP	pcGDP	The logarithm of per capita regional GDP.
	Secondary Industry Ratio	SIR	The proportion of the secondary industry’s added value in GDP.
	Population	Pop	The logarithm of the end-of-year resident population.
	GDP Growth	GDPgrowth	The growth rate of regional GDP from the previous year to the current year.

### 3.3 Descriptive statistics

As indicated in Table 2(A), the mean value of AEM is 0.064, suggesting widespread earnings management among listed companies in China. The sample includes 9.0% of firms with operating losses, 39.2% being state-owned enterprises, and 4.9% audited by the international Big 4 auditors. Additionally, the sample firms exhibit, on average, 16.0% sales growth, a 4.7% operating cash flow to total assets ratio, 42.4% financial leverage, and 37.2% of directors being independent. Notably, 21.1% of the sample firms are located in the pilot smart cities as categorized by the first method (Smart City). Conversely, 31.3% of the sample firms fall under the category of pilot smart cities as per the second definition method (Smart City*). Moreover, Table 2(B) illustrates that the average earnings management indicator for the treatment group is 0.061, lower than the control group’s mean of 0.065, with the difference being significant at the 1% level. This preliminary finding suggests that smart city construction may reduce earnings management among firms.

**Table 2:** Summary statistics.

**Variable**	**Mean**	**Std. Dev.**	**Minimum**	**Maximum**	**Observations**
(a) Descriptive Statistics for Full Samples					
AEM	0.064	0.061	0.001	0.441	21134^b^
Smart City	0.211	0.408	0	1	21134
Smart City*	0.313	0.376	0	1	21134
Size	22.163	1.213	19.178	27.072	21134
ListAge	2.146	0.759	0.693	3.296	21134
Lev	0.424	0.200	0.050	0.975	21134
CFO	0.047	0.065	-0.204	0.253	21134
Growth	0.160	0.344	-0.643	3.348	21134
SOE	0.392	0.488	0	1	21134
Loss	0.090	0.286	0	1	21134
Top1	0.347	0.140	0.085	0.748	21134
CIO	0.022	0.082	0	0.543	21134
CR10	58.295	14.230	22.450	95.990	21134
BSize	2.152	0.166	1.792	2.708	21134
BInd	0.372	0.050	0.333	0.571	21134
Meetings	2.194	0.363	1.386	3.178	21134
ExePay	14.359	0.660	12.490	16.432	21134
Dual	0.262	0.439	0	1	21134
Big4	0.049	0.217	0	1	21134
Opin	0.978	0.145	0	1	21134
pcGDP	10.779	0.601	8.463	13.156	2554
SIR	46.931	10.798	11.700	89.750	2554
Pop	5.848	0.831	1.366	8.136	2554
GDPgrowth	9.716	7.865	-20.630	61.851	2554
**Variable**	**Smart Cities (Treatment Group)**		**Normal Cities (Control Group)**		**Mean Difference**
	**Mean**	**Std. Dev.**	**Mean**	**Std. Dev.**	
(b) Univariate T-test for Firms from Smart Cities and Normal Cities					
AEM	0.061	0.061	0.065	0.061	-0.004***^a^
Observations	4459		16675		

^a^*** denotes significance levels at the 1%.

^b^The time range of the sample is from 2010 to 2020. Unless otherwise specified, the same applies to the following tables and figures.

Additionally, the correlation coefficients for all variables in the study are presented in S1 Table. The majority of independent variables and control variables demonstrate a correlation with AEM at 1% significance level. Consistent with expectations, Smart City is negatively correlated with AEM, significant at the 1% level. This finding supports Hypothesis H1, suggesting that smart city construction curbs corporate earnings management and improves the quality of accounting information.

## 4 Research results

### 4.1 The impact of smart city pilots on corporate earnings management

Table 3 presents the influence of smart city pilots on corporate earnings management. The first and second columns of Table 3 illustrate regression results based on two distinct definitions of smart cities (Smart City and Smart City*), respectively. Additionally, the sample period of the study spans from 2010 to 2020. The dependent variable is AEM, with control variables including firm-level controls, city-level controls, city-fixed effects, industry-fixed effects and year-fixed effects. Standard errors are adjusted for city-level clustering [64]. In subsequent regression analyses, both the control variables and clustered standard errors are consistently maintained. The baseline regression results are in line with our expectations. As shown in the second column of Table 3, smart city pilots lead to a decline of 0.006 in the earnings management of local firms, statistically significant at the 1% level. Given that the average AEM value among Chinese listed companies is 0.064, this indicates that the impact of smart city construction on earnings management is statistically and economically significant. This supports our research hypothesis H1, demonstrating that smart city pilot programs have a significant policy impact.

**Table 3:** Smart city pilots and firm earnings management.

Variable	(1) AEM–Smart City	(2) AEM–Smart City*
Smart City(Smart City*)×post	-0.006***^a^	-0.006***
	(0.002)^b^	(0.002)
Size	-0.004***	-0.004***
	(0.001)	(0.001)
ListAge	-0.002*	-0.002*
	(0.001)	(0.001)
Lev	0.008**	0.008**
	(0.004)	(0.004)
CFO	-0.185***	-0.185***
	(0.010)	(0.010)
Growth	0.016***	0.016***
	(0.002)	(0.002)
SOE	-0.004***	-0.004***
	(0.001)	(0.001)
Loss	0.029***	0.029***
	(0.002)	(0.002)
Top1	-0.006	-0.006
	(0.005)	(0.005)
CIO	-0.009**	-0.009**
	(0.004)	(0.004)
CR10	0.000***	0.000***
	(0.000)	(0.000)
BSize	-0.008***	-0.008***
	(0.003)	(0.003)
BInd	-0.002	-0.002
	(0.010)	(0.010)
Meetings	0.006***	0.006***
	(0.001)	(0.001)
ExePay	0.006***	0.006***
	(0.001)	(0.001)
Dual	-0.001	-0.001
	(0.001)	(0.001)
Big4	-0.001	-0.001
	(0.002)	(0.002)
Opin	-0.013***	-0.013***
	(0.003)	(0.003)
pcGDP	-0.002	-0.002
	(0.002)	(0.002)
SIR	0.000	0.000
	(0.000)	(0.000)
Pop	-0.005**	-0.005**
	(0.002)	(0.002)
GDPgrowth	0.000	0.000
	(0.000)	(0.000)
Constant	0.145***	0.146***
	(0.036)	(0.037)
Time Fixed Effects	Yes	Yes
City Fixed Effects	Yes	Yes
Industry Fixed Effects	Yes	Yes
Observations	21134	21134
R^2^	0.144	0.144

^a^*, **, and *** denotes significance levels at the 10%, 5%, and 1%, respectively.

^b^The regression model incorporates city-level clustered robust standard errors. Unless otherwise specified, the same applies to the following tables.

Regarding the control variables, both firm size and age show a negative correlation with earnings management, indicating that larger and older firms are less inclined to engage in earnings management, consistent with the findings of Cho et al. [53] and Bouaziz et al. [54]. Loss exhibits a significantly positive coefficient, aligning with prior studies and confirming that firms with operating losses are more motivated to manipulate earnings [65]. Leverage is positively correlated with earnings management, in line with previous findings that highly leveraged firms may be more driven to manage earnings to avoid debt covenant violations [40]. Cash flow from operations has a negative relationship with earnings management, corroborating Kothari et al.’s [55] findings. Additionally, sales growth has a positive association with earnings management, confirming the findings as identified by Collins [56]. An unqualified audit opinion is negatively correlated with earnings management, as evidenced by previous studies [63].

Furthermore, aligning with prior research [58], our results show that state-owned enterprises are less inclined towards earnings manipulation. Firms with common institutional investors tend to engage less in earnings manipulation [41], and those with larger boards appear to mitigate earnings management [59,60]. Conversely, firms with a higher ownership concentration demonstrate a positive correlation with earnings management [52,57], and increased executive compensation is associated with more earnings management [61]. Lastly, other firm-level variables like shareholding percentages of the largest shareholders, board independence, CEO duality, Big4, and city-level variables such as GDP per capita, GDP growth rate, and the secondary industry’s share in local GDP, exhibit nonsignificant coefficients, indicating these factors have a minimal impact on earnings manipulation.

### 4.2 Parallel trends test

This study employs a multi-period difference-in-differences (DID) model to analyze the impact of smart city pilots on corporate earnings management. Baseline regression results indicate that smart city pilots significantly reduce corporate earnings management. In order to ensure the reliability of the findings, an event study methodology is adopted to test the parallel trends assumption and examine the dynamic effects of smart city construction. The specific model is presented in Eq (5).

$$Y_{ijct}=\beta_{0}+\beta_{k}\sum_{k\geq -4}^{8}D_{t_{c0}+k}+\beta_{2}X_{ijct}^{\prime }+u_{j}+u_{c}+u_{t}+\varepsilon_{ict}$$ (5)

Where D_tc0+k_ indicates a group of dummy variables, with the subscript t_c0_+k representing the year in which city c implements the smart city pilot. In case t—t_c0_ = k, D_tc0+k_ equates to 1; otherwise, it equals 0. The year prior to the implementation of the smart city pilot policy (k = -1) is the omitted reference group in Eq (5). For instance, for the initial 2012 batch of pilot smart cities, -4, -3, and -2 represent the years 2008, 2009, and 2010, respectively, with 2011 being the reference group. This pattern is applied to subsequent batches, where the reference group is the year before the pilot’s commencement. Moreover, the set of parameters β_k_ in the model captures the influence of the smart city pilot policy in the kth year on corporate earnings management, relative to the reference year.

**Fig 1:**
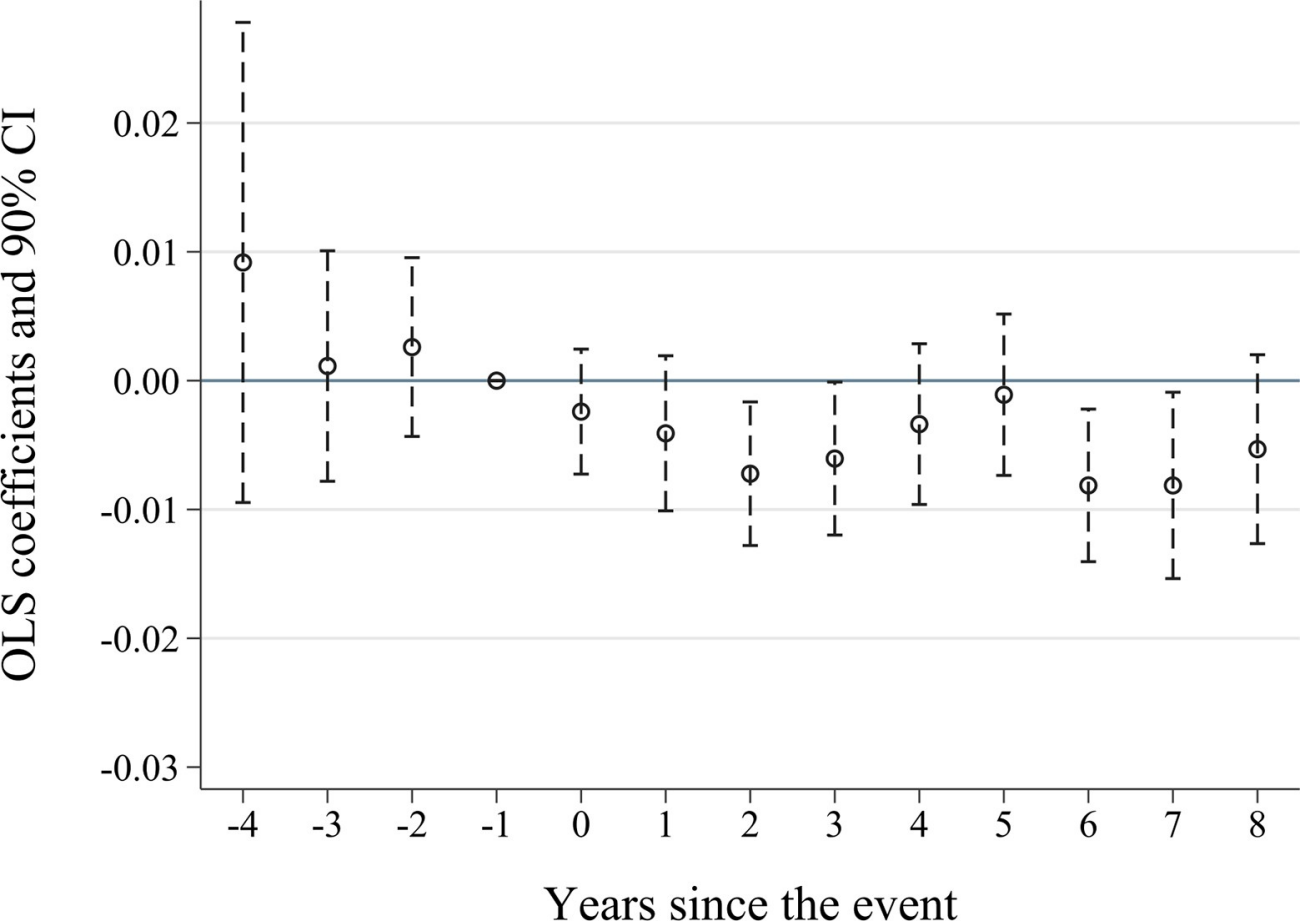
Parallel trend test.

Fig 1 presents the results of the parallel trends test. The coefficients of β_k_ are not significantly different from 0 in the 2nd, 3rd, and 4th years before the policy pilot. This indicates that there are no significant differences between the treatment and control groups prior to the smart city pilots, supporting the parallel trends assumption. Notably, in 4 of the 9 years following the smart city policy implementation, the treatment group exhibits significantly lower levels of earnings management compared to the control group, suggesting a significant and persistent effect of the policy.

### 4.3 Robustness test and additional analysis

#### 4.3.1 Robustness test

(1) Exclusion of Specific City Samples. To enhance the robustness of its findings, the study refined its city samples by excluding atypical cases. Initially, the study hypothesizes that the unique "corps-city integration" management model of the Xinjiang Production and Construction Corps significantly differs from standard city models, leading to their exclusion. Second, the study removed provincially-administered counties and county-level cities due to their lower administrative status and potential limitations in policy implementation, relative to prefecture-level cities. Subsequently, firms in autonomous prefectures and leagues, representing minority ethnic regions, were omitted. Table 4(A) displays regression results post the exclusion of these specific samples. Moreover, the study excludes the four centrally-administered municipalities due to their significant industrial agglomeration and resources and talent concentration, providing advantages not available in typical cities. Table 4(B) presents the regression results after this exclusion. The estimated coefficients for smart city pilots across all models remain statistically significant and consistent in magnitude and direction with those in Table 3, thereby affirming the robustness of the baseline regression outcomes.

**Table 4:** Robustness test.

Variable	(1) AEM–Smart City	(2) AEM–Smart City*
(a) Excluding of Samples from Leagues, Autonomous Prefectures, Provincially-Administered counties and county-level cities, and the Xinjiang Production and Construction Corps^b^		
Smart City(Smart City*)×post	-0.006***^a^	-0.006***
	(0.002)	(0.002)
Observations	20889	20889
R2	0.142	0.142
(b) Excluding of Samples from the Four Centrally-Administered Municipalities		
Smart City(Smart City*)×post	-0.006***	-0.005**
	(0.002)	(0.002)
Observations	17182	17182
R2	0.145	0.145
€ Sample Period Extended from 2007 to 2020		
Smart City(Smart City*)×post	-0.004**	-0.004**
	(0.002)	(0.002)
Observations	23886	23886
R2	0.134	0.134
(d) Additional Control for Green Credit Policy		
Smart City(Smart City*)×post	-0.006***	-0.006***
	(0.002)	(0.002)
Green Credit×post*	0.004	0.004
	(0.005)	(0.005)
Observations	21133	21133
R2	0.144	0.144
(e) Additional Control for Low-Carbon City Pilot		
Smart City(Smart City*)×post	-0.006***	-0.006***
	(0.002)	(0.002)
Low-Carbon City×post*	-0.000	-0.000
	(0.001)	(0.001)
Observations	21133	21133
R2	0.144	0.144
(f) Estimation Results Utilizing the PSM-DID Method		
Smart City(Smart City*)×post	-0.005**	-0.005**
	(0.002)	(0.002)
Observations	12053	12053
R2	0.154	0.154

^a^** and *** denotes significance levels at the 5% and 1%, respectively.

^b^We control for time-fixed effects, industry-fixed effects, and city-fixed effects in all regressions.

**Fig 2:**
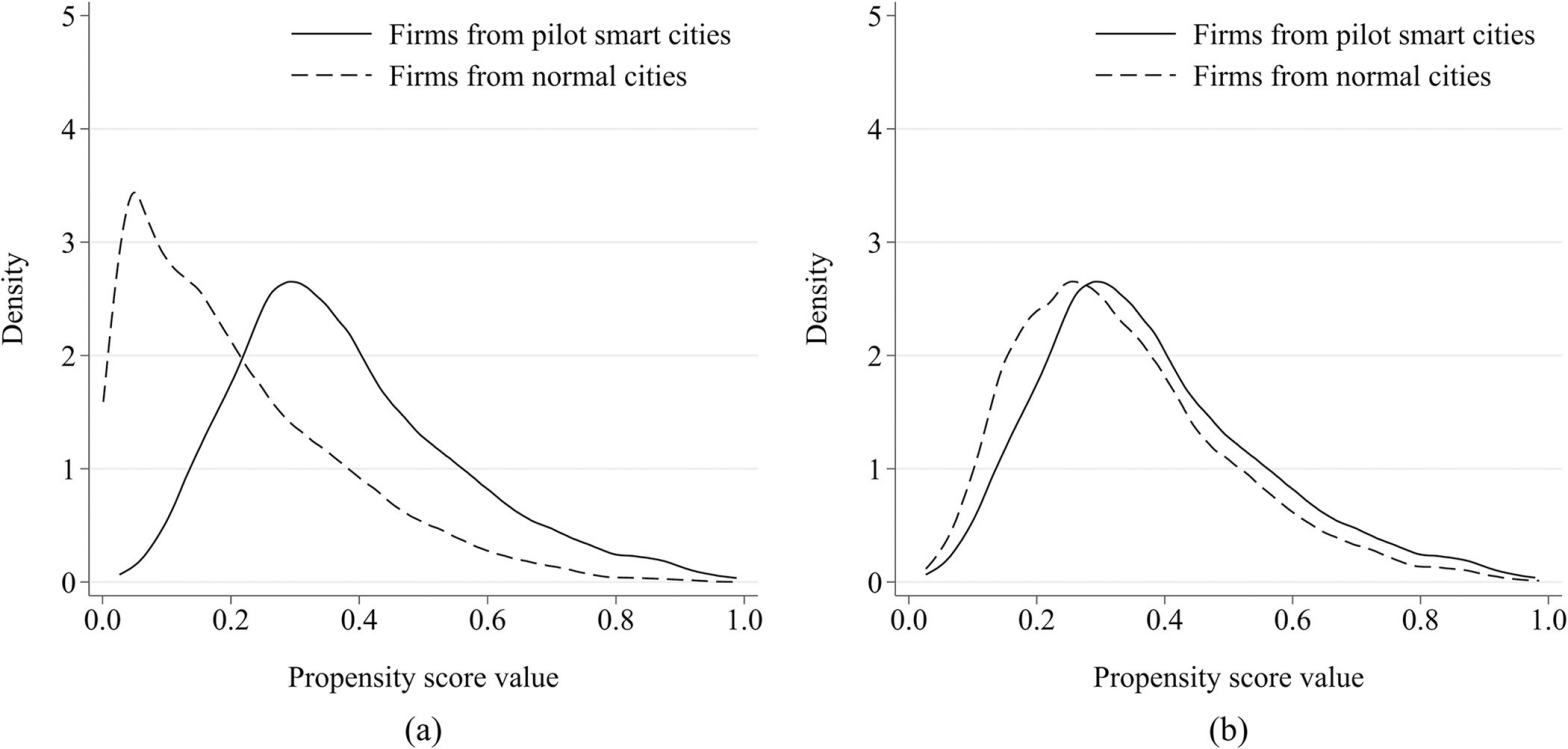
Distribution of propensity scores before and after matching for pilot smart cities. (a) Distribution of propensity scores before matching for pilot smart cities. (b) Distribution of propensity scores after matching for pilot smart cities.

**Fig 3:**
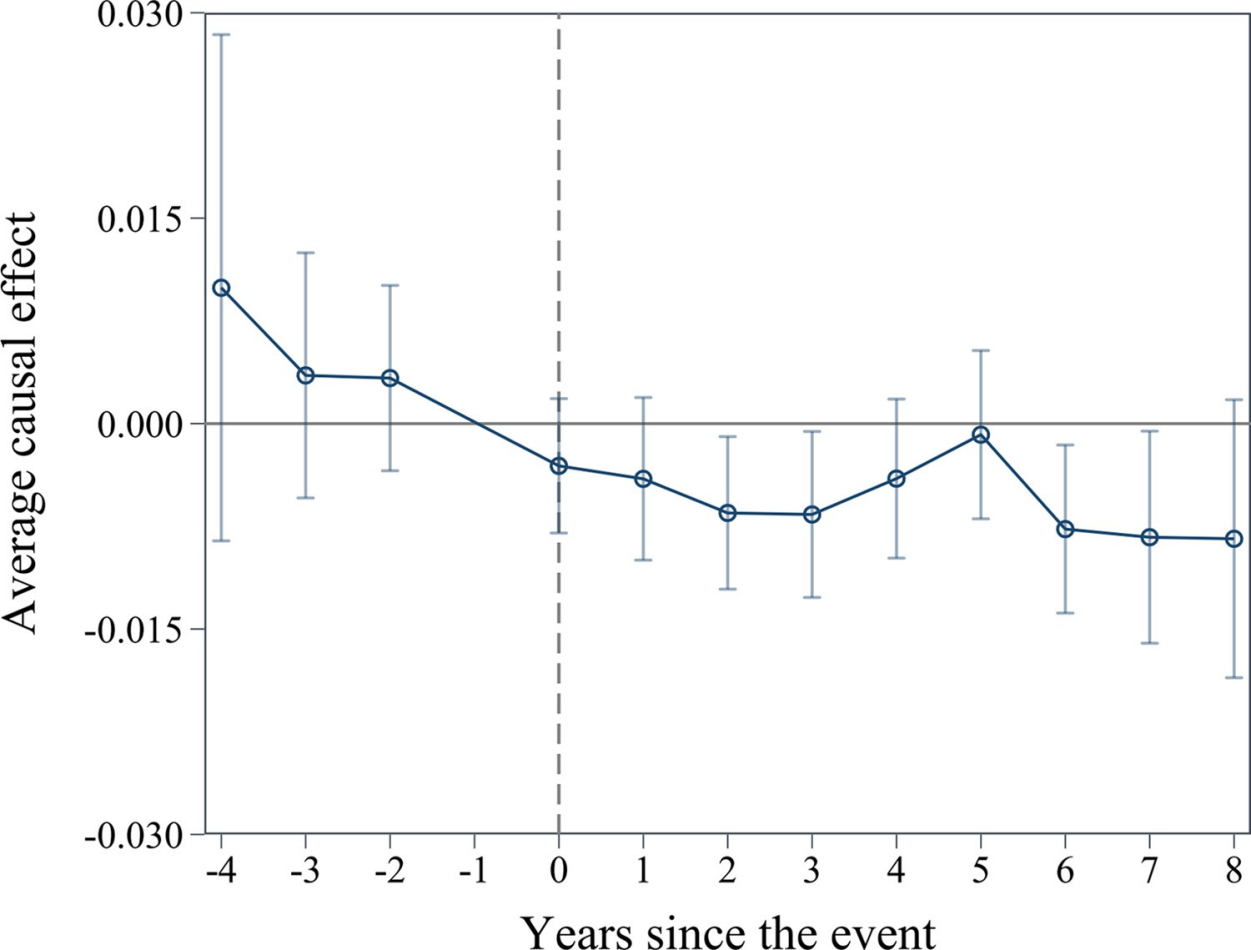
Sun and Abraham approach.

**Fig 4:**
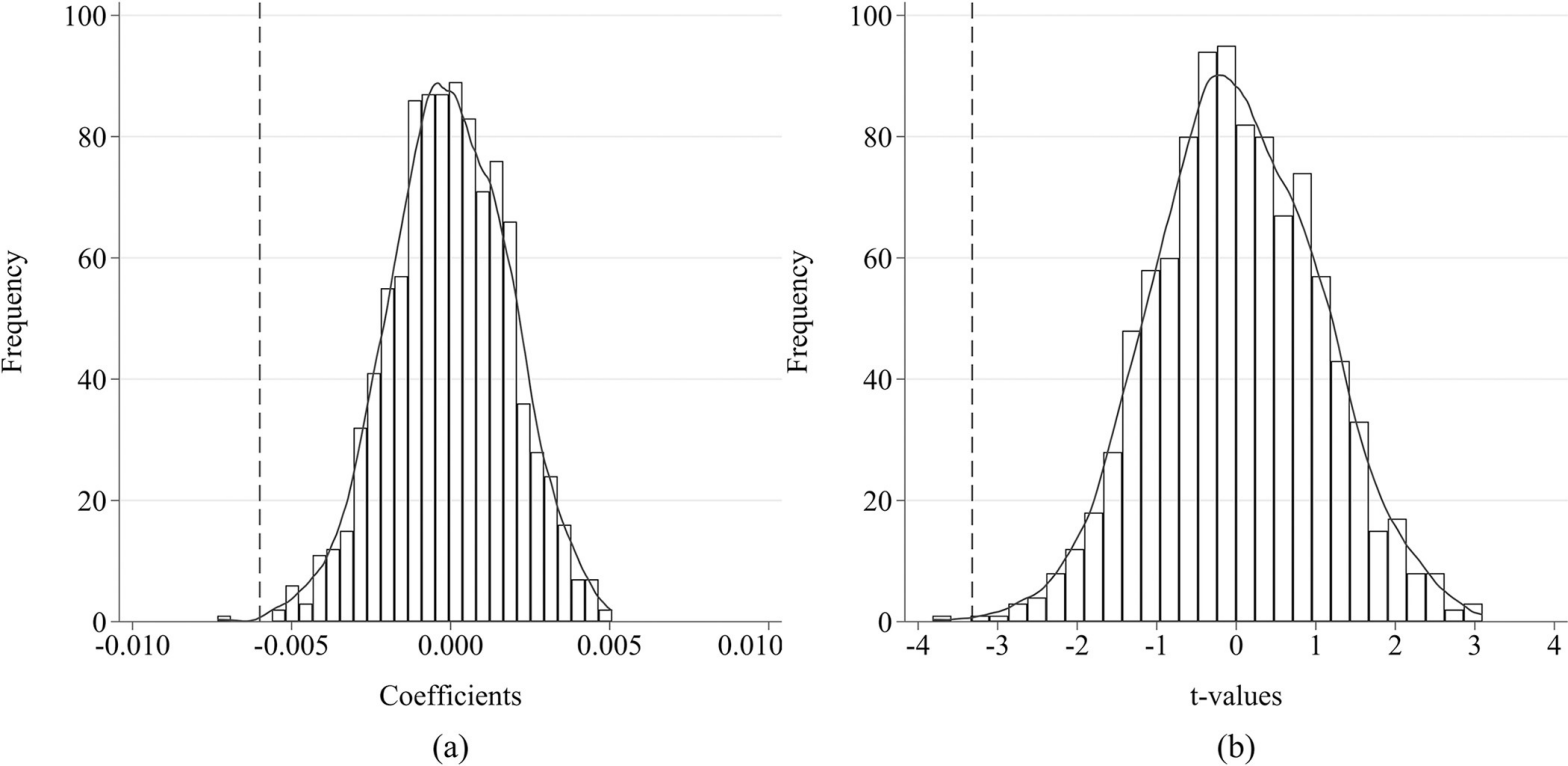
The results of the placebo test. (a) Distribution of coefficients. (b) Distribution of t-values.

(2) Extending the Sample Period. The smart city pilot program was primarily implemented from 2012 to 2014, while the baseline regression analysis in this study encompasses a sample period from 2010 to 2020. For the last batch of smart city pilot projects, the sample covered four years prior to the policy impact, a duration that may not suffice for robust research conclusions. Consequently, the sample period has been extended back to 2007. Regression results are detailed in Table 4(C). The corresponding coefficients are significantly negative, reinforcing the robustness of the baseline regression estimates.

(3) Isolating policy effects. Additional dummy variables representing the “green credit policy” and the “low-carbon city pilot” were incorporated to mitigate the impact of concurrent policies. The corresponding regression results are displayed in Panels (d) and (e) of Table 4. It is clear that both the magnitude and direction of the coefficients are consistent with the baseline regression results; the impact of smart city construction on earnings management is significantly negative, thereby affirming the robustness and consistency of the conclusion.

(4) Implementing the PSM-DID model. To mitigate potential endogeneity, the study initially employs a Logit model to explore the factors affecting a city becoming a smart city pilot unit. Thereafter, propensity score matching is undertaken to eliminate sample selection bias. Following this, the DID analysis, utilizing the matched sample, assesses the impact of smart city pilots on corporate earnings management. Fig 2 depicts the kernel density distribution of propensity scores obtained through 1:3 nearest neighbor matching, with Smart City as the dependent variable, using control variables consistent with the baseline regression. Notably, there is considerable overlap in propensity scores between the matched treatment and control groups, indicating effective matching. The regression analysis with the matched sample, presented in Table 4(F), reveals that smart city pilots significantly reduce earnings management, further reinforcing the robustness of our baseline regression findings.

(5) Sun and Abraham [66] approach. In China, the smart city pilot program was implemented in a staggered manner. This approach leads to heterogeneity across cities and over time, which may cause bias of our two-way fixed effects (TWFE) estimator. To address this issue, we employed the Sun and Abraham (SA) approach. This method enables control over the heterogeneous treatment effects among cohorts to accurately assess the impact of smart city pilot. The results of the SA approach are visually depicted in Fig 3. The coefficients of interest are not significantly different from 0 in the 2nd, 3rd, and 4th years before the policy pilot. However, in the 2nd, 3rd, 6th and 7th years following the implementation of the smart city pilot, the treatment group demonstrates significantly lower levels of earnings management compared to the control group. These findings suggest that our baseline regression has not suffered from significant bias due to heterogeneous treatment effects across units, ensuring the robustness of our results.

(6) Placebo test. A placebo test is conducted to ensure that the significant impact of the smart city pilot on earnings management is not attributed to random factors. Specifically, a "treatment group" and a "control group" are simulated by randomly selecting cities to join the smart city pilot program. The number of cities simulated to join the smart city pilot each year is the same as in reality. Subsequently, a regression analysis is carried out using these simulated smart cities. This process is repeated 1000 times to obtain 1000 sets of regression coefficients and t-values. The distribution of these coefficients and t-values is illustrated in Fig 4. The kernel density estimates of the simulated coefficients and t-values are centered around zero (0) and significantly deviate from the coefficients and t-statistics measured by the baseline model under the actual "smart city pilot" policy shock (represented by vertical dashed lines in Fig 4(A) and 4(B)). Furthermore, the coefficients derived by the baseline model fall outside two standard deviations (SDs) of the distribution of the simulated estimation results. These findings confirm that no statistically significant impact of the smart city pilot is observed for the simulated treatment group, thereby affirming the robustness of the earlier estimated results.

#### 4.3.2 Additional analysis

(1) Substitution between AEM and real earnings management (REM). Evidence from prior studies suggests that firms use accrual and real earnings management strategies as substitutes in managing earnings [67,68]. Therefore, smart city construction may influence corporate earnings management, possibly causing a shift from accrual-based to real earnings management. To test this hypothesis, the study first evaluates the impact of smart city construction on REM, and then estimates the impact of smart city construction on accrual-based earnings management while controlling for REM. If a substitution effect exists between AEM and REM, two outcomes are expected: First, an increase in real earnings management (REM) attributable to smart city construction. Second, when controlling for REM, the influence of smart city construction on accrual-based earnings management (AEM) significantly reduces, and the estimated coefficient of REM on AEM is significantly negative. Regression analysis results, presented in Table 5(A), indicate that smart city construction has no significant impact on REM. Additionally, the correlation coefficient between real earnings management and accrual-based earnings management is not significant. As a result, our hypothesis is not supported by these findings.

**Table 5:** Additional analysis.

(a) The Impact on Real Earnings Management^b^		
Variable	(1) REM–Smart City	(2) AEM–Smart City
Smart City×post	-0.004	-0.004**^a^
	(0.007)	(0.002)
REM		0.001
		(0.004)
Observations	19654	19303
R2	0.433	0.136
(b) The Impact on the Direction of Earnings Manipulation		
	(1) Positive Group	(2) Negative Group
Smart City×post	-0.005**	-0.005
	(0.002)	(0.004)
Observations	12266	8868
R2	0.356	0.303

^a^** denotes significance levels at the 5%.

^b^We control for time-fixed effects, industry-fixed effects, and city-fixed effects in all regressions.

(2) The direction of earnings management. This section investigates the direction of earnings management that is more likely to be affected by smart city construction. Following Jiang et al.’s [52] approach, samples were categorized into two groups based on whether the discretionary accruals (DA) were positive. The results, shown in Panel (b) of Table 5, demonstrate that the coefficient for smart city construction is significantly negative in the group with positive DA. This indicates that firms in smart cities are less inclined towards positive earnings manipulation. A possible explanation is that improvements in the external information environment, attributed to smart city construction, induce a more conservative approach among executives, prompting them to adopt a more cautious financial policy.

## 5 Underlying mechanism and heterogeneity analysis

### 5.1 Underlying mechanism

This section explores the mechanisms through which smart city construction impacts corporate earnings management. Referring to the methods used in previous studies [69], this study conducts an economic mechanism analysis using Eqs (6) and (7).

$${Mediator}_{ijct}=\beta_{0}+\beta_{1}∙{Smart\;City}_{ct}∙{post}_{t}+\beta_{2}∙{Smart\;City}_{c}+\beta_{3}∙{post}_{t}+\beta_{4}X_{ijct}^{\prime }+u_{j}+u_{c}+u_{t}+\varepsilon_{ijct}$$ (6)

$${AEM}_{ijct}=\beta_{0}+\beta_{1}∙{Smart\;City}_{ct}∙{post}_{t}+\beta_{2}∙{Mediator}_{ijct}+\beta_{3}∙{Smart\;City}_{c}+\beta_{4}∙{post}_{t}+\beta_{5}X_{ijct}^{\prime }+u_{j}+u_{c}+u_{t}+\varepsilon_{ijct}$$ (7)

Where Mediator_ijct_ represents the economic mechanism variable. Control variables and clustered standard errors remain consistent with the baseline regression. Based on hypothesis H2, this research tentatively infers that the smart city pilot impacts earnings management through the channels of improving the local information environment.

The 2012 "National Smart City (District, Town) Pilot Index System" mandates that pilot cities enhance their communication and network infrastructure, and establish both public information platforms and fundamental city databases [21]. This advancement in smart city construction is expected to significantly enhance the local information environment, thereby reducing information asymmetry. Following methodologies from previous research [70], this study utilizes the volume-synchronized probability of informed trading (VPIN) indicator as a proxy variable for the information environment. VPIN is calculated as the ratio of the arrival rate of information-based trading orders to the total arrival rate of all orders, as shown in Eq (8):

$$\left\{ \begin{array}{c} V_{\tau }^{B}=\sum_{i=t(\tau -1)+1}^{t(\tau )}V_{i}∙Z(\frac{P_{i}-P_{i-1}}{\sigma_{\Delta P}})\\ V_{\tau }^{S}=\sum_{i=t(\tau -1)+1}^{t(\tau )}V_{i}∙[1-Z(\frac{P_{i}-P_{i-1}}{\sigma_{\Delta P}})]\\ VPIN=\frac{\sum_{\tau =1}^{n}\left|V_{\tau }^{B}-V_{\tau }^{S}\right|}{nV} \end{array}\right.$$ (8)

Where t(τ) denotes the final time limit for the τ trading basket; i indicates the smallest time interval, which is set as 1 minute in this paper. V_i_ represents the trading volume at time i, and P_i_ represents the price at the time i. Moreover, σ_Δp_ denotes the standard deviation of price changes for all baskets. Z stands for the cumulative distribution function of the standard normal distribution. Furthermore, n refers to the number of trading baskets within the time interval, with 50 baskets estimated per day in this study. V signifies the equalized trading volume for each basket. Lastly, the daily VPIN values are averaged to derive the yearly VPIN value. Assuming smart city construction enhances the local information environment, a reduction in VPIN should be observable in the pilot smart cities.

The potential influence of smart city pilots on the local information environment is estimated by utilizing VPIN as the dependent variable and employing Eq (6) for regression analysis. Subsequently, Eq (7) is used to assess the impact of smart city construction on accrual-based earnings management (AEM), while controlling for VPIN. The outcomes of the regression analysis are presented in Table 6. The results demonstrate that the regression coefficient of the smart city pilot on the probability of informed trading is significantly negative. This suggests that smart city construction decreases the probability of informed trading, thus reducing market information asymmetry and notably improving the local information environment. Furthermore, the regression results shown in the second column of Table 6 indicate that local information environmental partially mediates the impact of smart city construction on corporate earnings management. These findings highlight that smart city construction significantly improves the local information environment, increasing the challenges associated with earnings management and deterring such practices [39]. Consequently, these results affirm hypothesis H2 of the study.

**Table 6:** Mechanism test.

Variables	(1) VPIN	(2) AEM
Smart City×post	-0.005***^a^	-0.006***
	(0.002)	(0.002)
VPIN		0.106***
		(0.021)
Time, City, and Industry Fixed Effects	Yes	Yes
Observations	20831	20831
*R* ^2^	0.454	0.145

^a^*** denotes significance level at the 1%.

^b^Parallel trend tests showed no significant differences between treatment and control groups before policy implementation, satisfying the parallel trends assumption.

### 5.2 Heterogeneity analysis

#### 5.2.1 Heterogeneity in regions

**Fig 5:**
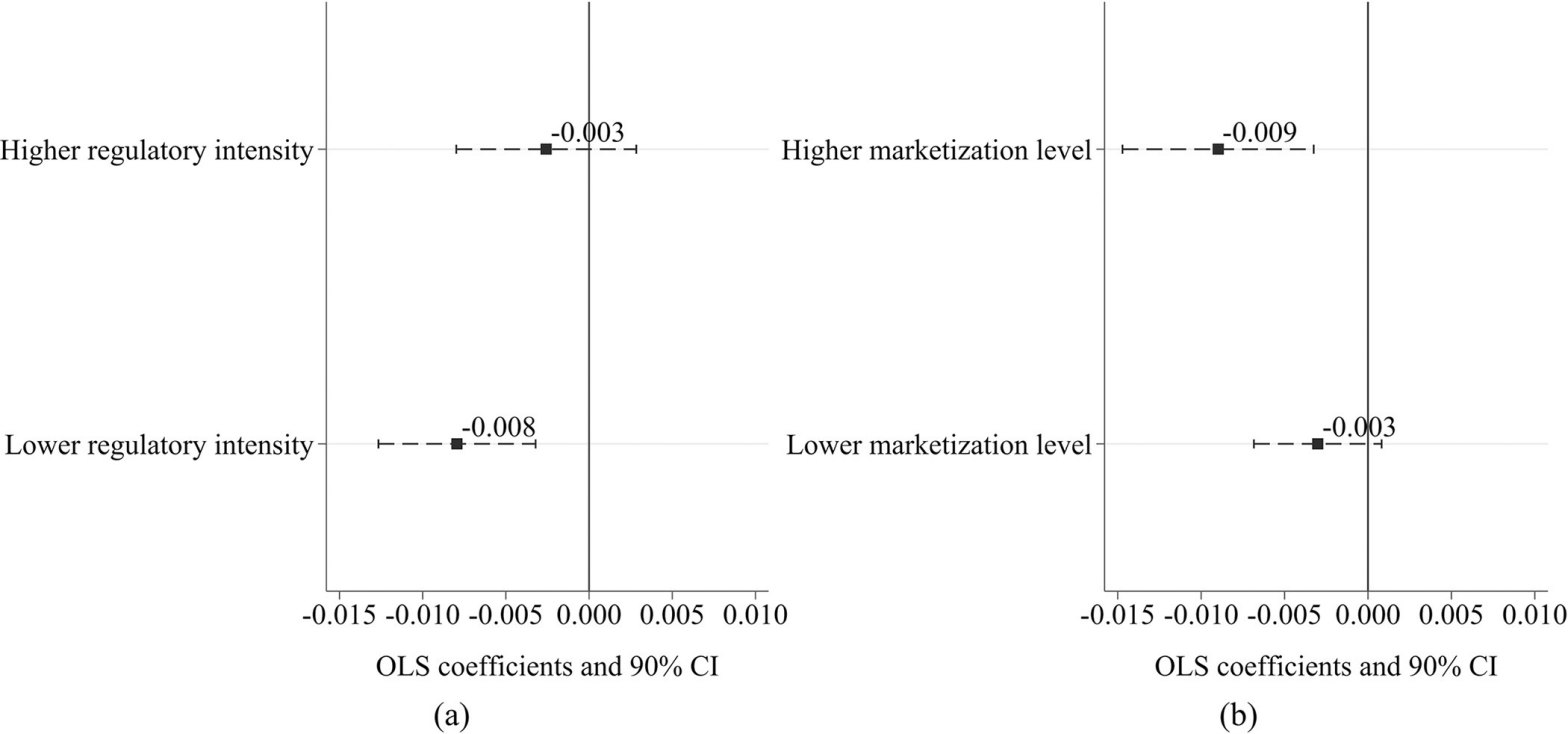
Regulatory intensity, marketization level, and corporate earnings management. (a) Heterogeneity in regulatory intensity. (b) Heterogeneity in marketization level.

Given the considerable regional variations in regulatory intensity across China, the impact of smart city construction on corporate earnings management may differ by region. To test this hypothesis, the study uses the burden of government regulation, a sub-index of the China Marketization Index, to measure local government regulation intensity. The research sample is categorized into two groups based on the median value of this index [71]. Subsequently, Eq (1) is used to conduct separate regression analyses for each group, and the results are presented in Fig 5(A). In the group with lower regulatory intensity, smart city pilots significantly reduced corporate earnings management by 0.008, and the influence is statistically different from 0 at the 1% level of significance. However, in regions with higher regulatory intensity, the influence of smart city pilots on earnings management is not significant. Detailed regression results in this section are provided in S2 Table for reference.

Furthermore, this section explores how the impact of smart city pilots on corporate earnings management differs across regions with varying levels of marketization. Utilizing the China Marketization Index, the research samples are divided into two subgroups based on their median values. Subsequently, separate regression analyses are performed for each subgroup. The results, as presented in Fig 5(B), indicate that smart city pilots significantly affect corporate earnings management in regions with higher marketization levels. Conversely, in regions with lower marketization levels, the impact of smart city initiatives on corporate earnings management appears to be insignificant.

#### 5.2.2 Heterogeneity in industries

**Fig 6:**
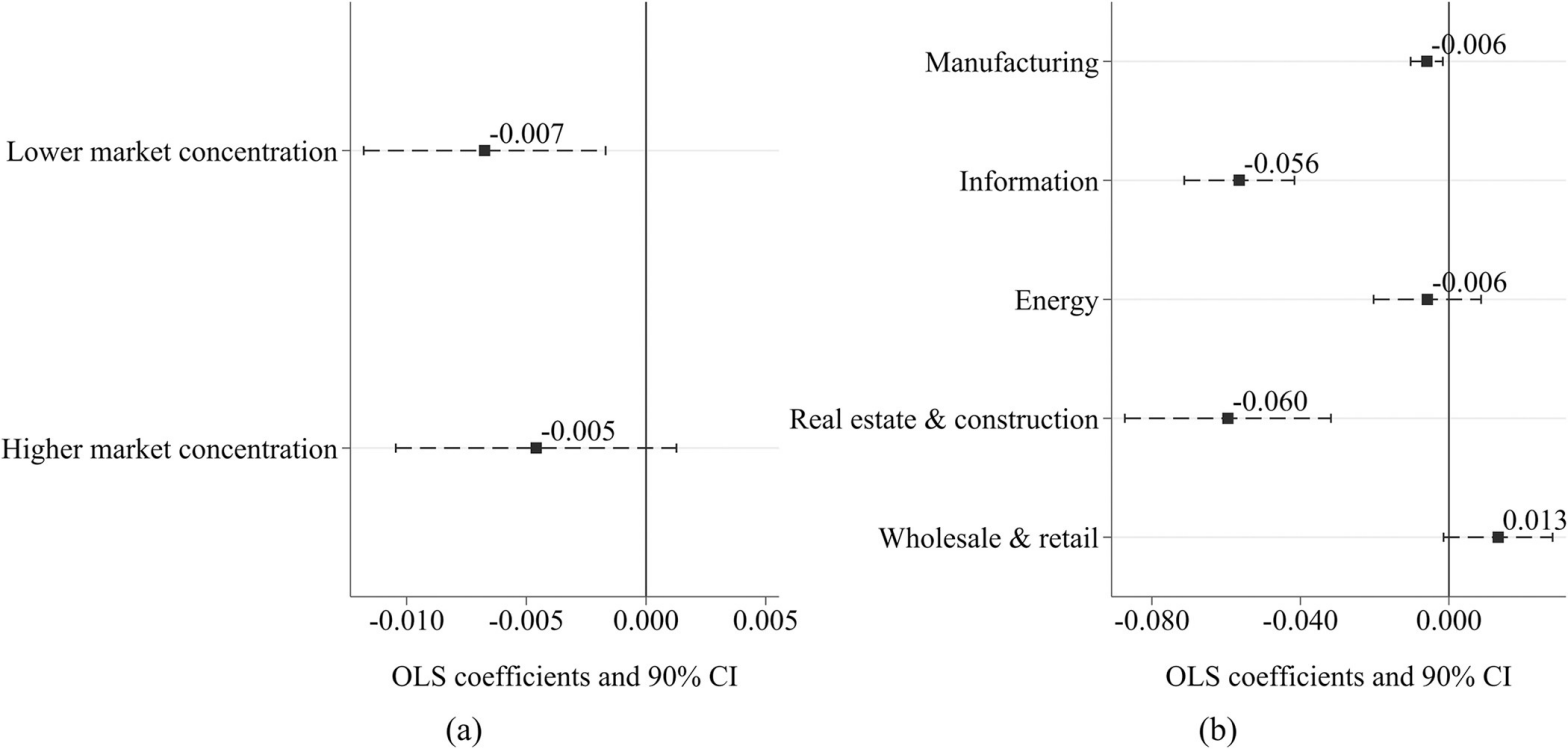
Industry characteristics and corporate earnings management. (a) Heterogeneity in market concentration. (b) Heterogeneity across industries.

As China’s market economy evolves, notable differences in market concentration among various industries have emerged, suggesting that the impact of smart city construction on corporate earnings management may differ across industries. To examine this hypothesis, this study categorizes the research samples into two subsamples based on the median value of the industry’s Herfindahl-Hirschman Index (HHI) and carries out separate regression analyses for each group using Eq (1). The regression findings, displayed in Fig 6(A), support the aforementioned research hypothesis. In industries with lower market concentration, smart city pilot projects significantly reduce corporate earnings management. However, in industries with higher market concentration, the effect of these projects is not statistically significant.

Moreover, a detailed analysis of industry heterogeneity reveals that smart city pilot projects exert a stronger inhibitory influence on earnings management in the real estate and construction industries, as well as in the information transmission, software and information technology service sectors. As illustrated in Fig 6(B), the policy impact of smart city pilots results in a 0.060 reduction in earnings management in the real estate and construction industries and a 0.056 decrease in the information transmission, software, and information technology service sectors. Both changes are statistically significant at the 1% level. Additionally, in the manufacturing industry, the policy effect on earnings management is -0.006, significantly different from 0 at the 5% level. However, in the energy sector and in retail and wholesale industries, the policy impact on earnings management is not statistically significant.

## 6 Discussion

This study investigates the influence of smart city pilots on the earnings management of China’s listed companies for the time period from 2010 to 2020, exploring the underlying mechanisms of the proposed influence. The findings suggest that smart city construction significantly inhibits corporate earnings management. Further analysis reveals that smart city pilots mainly affect earnings management by improving the external information environment, thus curbing earnings management practices.

Corporate earnings management is often associated with increased managerial opportunism and issues of asymmetric information, leading to underinvestment and overinvestment problems [72]. Therefore, developing strategies to mitigate corporate earnings management remains a key research focus for scholars. Previous literature suggests that factors including social trust [73], industry competition level [74], corporate governance structure [75], lead independent directors [76], and fair value measurements [77] influence corporate earnings management. This paper proposes that the construction of smart cities can effectively reduce corporate earnings management, offering a new approach to its mitigation.

In the heterogeneity analysis section, this study initially examines the diverse impacts across various geographic regions. The study reveals that in regions with lower regulatory intensity and in areas with higher marketization levels, smart city pilots are notably more effective in curbing corporate earnings management. This underscores the complementary role of smart city construction to existing government regulation and the enhancing effect of higher marketization levels on the efficacy of smart city construction [78].

Apparently, in regions with lower regulatory intensity, the risk of earnings management detection is typically lower, providing greater incentives for internal actors to engage in such practices [79,80]. Similarly, more marketized provinces tend to provide less oversight and exhibit higher tolerance for earnings management [81]. These conditions generally lead to increased earnings management in local firms, while also offering significant potential for reduction. Consequently, it aligns with our expectations that smart city construction has a more pronounced inhibitory effect on corporate earnings management in regions with lower regulatory intensity and higher marketization levels [82]. This implies that smart city construction could be an effective alternative to traditional government regulation.

Furthermore, this research explores the varied impacts of smart city construction on earnings management across different industries. The findings indicate that smart city construction has a more pronounced impact on earnings management in less concentrated markets.

Previous research has found that firms operating in concentrated markets use more accrual and real earnings management compared to those in less concentrated markets [74]. This phenomenon can be attributed to two reasons. Firstly, reduced competition in these markets lessens the probability of firms providing accurate disclosures to investors, which in turn decreases the detection of companies involved in account manipulation [83]. Secondly, in concentrated markets, limited competition for financial resources decreases the necessity for comprehensive information disclosure aimed at reducing capital costs [84]. Consequently, financial malpractices are prevalent in concentrated markets, raising concerns about the effectiveness of governance mechanisms in preventing accounting manipulation in these industries [74]. Similarly, this study finds that smart city construction fails to curb corporate earnings management in highly concentrated industries, potentially exacerbating concerns about earnings management in these sectors.

Furthermore, the study reveals that in industries closely associated with smart city development, the impact of smart city construction on corporate earnings management is more pronounced. Specifically, smart city pilots stimulate significant growth in regional digital projects, resulting in substantial enhancements in the local information environment [10,16]. In addition, the optimization and transformation of urban environments through smart city construction enhances the attractiveness of cities to migrants, thereby increasing the local housing demand [85]. As the information, real estate, and construction industries are the primary beneficiaries of these policy dividends, they emphasize policy implementation and exhibit enhanced cooperation. These industries are more motivated and employ more systematic approaches to leverage the benefits of smart city initiatives, leading to a more substantial reduction in earnings management.

## 7 Conclusion and policy implications

The findings of this study suggest that smart city construction significantly reduces corporate earnings management by improving external information environments. This effect is more significant in regions with lower regulatory intensity and higher marketization levels, as well as in firms operating in less concentrated markets or those more closely aligned with smart city construction.

The results of this research carry significant policy implications. First, traditional approaches and external oversight may not always be effective in curbing corporate earnings management, owing to insufficient requisite expertise and experience. However, by promoting the construction of smart cities, regulatory authorities can more effectively reduce corporate earnings management, thereby optimizing the business information environment and mitigating information asymmetry, and thus contributing to the establishment of a fairer market.

Second, smart city construction can serve as a substitute for government regulation in reducing corporate earnings management. Consequently, with the global expansion of smart cities, there is an opportunity to further relax government regulations on enterprises, thereby fostering a more conducive environment for business development.

Third, the impact of smart city construction on corporate earnings management is less pronounced in industries with higher market concentration and those less associated with smart city development, compared to sectors with lower market concentration or a closer association with smart city initiatives. As smart cities evolve, the existing industry-specific heterogeneity may further exacerbate the disparities in earnings management across different sectors. These disparities could lead to systematic deviations in earnings reporting within certain industries, thereby causing inefficient resource allocation. The potential negative consequences of such a situation warrant managerial attention.

Lastly, this study has inherent limitations. Specifically, the policy impact of the smart city pilot, central to this research, occurred from 2012 to 2014. During this period, the concept of smart city construction was significantly different from the current interpretations. Consequently, the observed effects of smart city construction on corporate earnings management during this period may not completely coincide with its current attributes. Nonetheless, this project is unique, being the only nationwide smart city pilot in China and resembling a random policy shock. Analyzing the impact of smart city construction through this policy shock has produced robust research outcomes. Therefore, despite its limitations, this study highlights important findings on the factors influencing corporate earnings management and offers new insights into the broader economic implications of smart cities.

## Supporting information

S1 Table The correlation matrix of main variables. (DOCX)

S2 Table Regression results of heterogeneity analysis. (DOCX)
