# Supplementary material for: Smart city and earnings management: Evidence from China
Source: PLoS One. 2024 Apr 2;19(4):e0301025. doi: 10.1371/journal.pone.0301025 (PMC10986951; doi:10.1371/journal.pone.0301025)
Supplement: S1 Table — (DOCX) [file pone.0301025.s001.docx]

**Table S1.** The correlation matrix of main variables.

|  | AEM | Smart City | Smart City* | Size | ListAge | Lev | CFO |
| --- | --- | --- | --- | --- | --- | --- | --- |
| AEM | 1 |  |  |  |  |  |  |
| Smart City | -0.03***^a^ | 1 |  |  |  |  |  |
| Smart City* | -0.04*** | 0.95*** | 1 |  |  |  |  |
| Size | -0.06*** | 0.02*** | 0.03*** | 1 |  |  |  |
| ListAge | -0.03*** | 0.03*** | 0.03*** | 0.42*** | 1 |  |  |
| Lev | 0.06*** | -0.02** | -0.03*** | 0.50*** | 0.36*** | 1 |  |
| CFO | -0.23*** | 0.04*** | 0.04*** | 0.05*** | -0.01* | -0.17*** | 1 |
| Growth | 0.08*** | -0.03*** | -0.04*** | 0.02** | -0.11*** | 0.02*** | 0 |
| SOE | -0.04*** | -0.05*** | -0.07*** | 0.35*** | 0.48*** | 0.30*** | -0.02*** |
| Loss | 0.15*** | 0.01* | 0.01* | -0.05*** | 0.10*** | 0.16*** | -0.16*** |
| Top1 | -0.03*** | -0.02*** | -0.03*** | 0.18*** | -0.06*** | 0.05*** | 0.08*** |
| CIO | -0.05*** | -0.0100 | -0.0100 | 0.22*** | 0.15*** | 0.11*** | 0.03*** |
| CR10 | -0.02*** | -0.0100 | 0 | 0.10*** | -0.39*** | -0.12*** | 0.12*** |
| BSize | -0.03*** | 0.03*** | 0 | 0.24*** | 0.15*** | 0.14*** | 0.02*** |
| BInd | 0.0100 | -0.04*** | -0.03*** | 0.0100 | -0.03*** | -0.0100 | -0.0100 |
| Meetings | 0.08*** | -0.05*** | -0.05*** | 0.22*** | 0.05*** | 0.22*** | -0.14*** |
| ExePay | -0.04*** | -0.05*** | 0 | 0.40*** | 0.12*** | 0.07*** | 0.16*** |
| Dual | 0.01* | -0.0100 | 0 | -0.18*** | -0.26*** | -0.14*** | 0 |
| Big4 | -0.03*** | -0.06*** | -0.05*** | 0.30*** | 0.08*** | 0.10*** | 0.07*** |
| Opin | -0.07*** | 0 | -0.0100 | 0.02*** | -0.06*** | -0.09*** | 0.06*** |
| pcGDP | -0.03*** | -0.07*** | 0.05*** | 0.03*** | -0.08*** | -0.04*** | -0.02*** |
| SIR | 0.04*** | 0.21*** | 0.12*** | -0.10*** | -0.04*** | 0.02*** | 0.0100 |
| Pop | -0.03*** | -0.23*** | -0.11*** | 0.05*** | -0.01** | -0.01** | 0 |
| GDPgrowth | 0.07*** | -0.13*** | -0.22*** | -0.08*** | -0.04*** | 0.04*** | -0.05*** |
|  | Growth | SOE | Loss | Top1 | CIO | CR10 | BSize |
| Growth | 1 |  |  |  |  |  |  |
| SOE | -0.08*** | 1 |  |  |  |  |  |
| Loss | -0.20*** | 0.02*** | 1 |  |  |  |  |
| Top1 | 0 | 0.20*** | -0.08*** | 1 |  |  |  |
| CIO | -0.02** | 0.24*** | -0.0100 | 0.06*** | 1 |  |  |
| CR10 | 0.09*** | -0.08*** | -0.13*** | 0.59*** | 0.09*** | 1 |  |
| BSize | -0.02*** | 0.28*** | -0.02** | 0.02*** | 0.12*** | -0.0100 | 1 |
| BInd | 0 | -0.07*** | 0.0100 | 0.03*** | -0.0100 | 0.03*** | -0.55*** |
| Meetings | 0.13*** | -0.04*** | 0.02*** | -0.05*** | -0.01* | -0.02** | -0.04*** |
| ExePay | 0.03*** | 0 | -0.10*** | -0.02** | 0.08*** | 0.09*** | 0.05*** |
| Dual | 0.03*** | -0.31*** | -0.0100 | -0.04*** | -0.10*** | 0.06*** | -0.19*** |
| Big4 | -0.01* | 0.12*** | -0.02** | 0.11*** | 0.14*** | 0.14*** | 0.07*** |
| Opin | 0.07*** | 0.02** | -0.21*** | 0.05*** | 0.02*** | 0.06*** | -0.0100 |
| pcGDP | 0 | -0.15*** | -0.02** | -0.0100 | -0.0100 | 0.08*** | -0.14*** |
| SIR | 0.02** | -0.04*** | -0.01** | -0.02*** | -0.04*** | -0.04*** | 0.06*** |
| Pop | -0.02** | 0.04*** | -0.02*** | 0.01** | 0.06*** | 0.04*** | -0.0100 |
| GDPgrowth | 0.11*** | 0.10*** | -0.06*** | 0.05*** | 0.03*** | 0 | 0.09*** |
|  | BInd | Meetings | ExePay | Dual | Big4 | Opin | pcGDP |
| BInd | 1 |  |  |  |  |  |  |
| Meetings | 0.05*** | 1 |  |  |  |  |  |
| ExePay | 0.0100 | 0.12*** | 1 |  |  |  |  |
| Dual | 0.11*** | 0.01* | 0.03*** | 1 |  |  |  |
| Big4 | 0.03*** | 0.02*** | 0.18*** | -0.06*** | 1 |  |  |
| Opin | 0 | -0.02*** | 0.04*** | 0.01** | 0 | 1 |  |
| pcGDP | 0.06*** | 0.05*** | 0.28*** | 0.11*** | 0.04*** | 0.01* | 1 |
| SIR | -0.06*** | -0.09*** | -0.23*** | -0.0100 | -0.08*** | -0.01* | -0.31*** |
| Pop | 0.0100 | 0 | 0.15*** | 0.0100 | 0.08*** | 0.03*** | 0.20*** |
| GDPgrowth | -0.03*** | -0.0100 | -0.15*** | -0.03*** | 0 | 0.02*** | -0.14*** |
|  | SIR | Pop | GDPgrowth |  |  |  |  |
| SIR | 1 |  |  |  |  |  |  |
| Pop | -0.46*** | 1 |  |  |  |  |  |
| GDPgrowth | 0.18*** | -0.11*** | 1 |  |  |  |  |

^a^*, **, and *** denote that correlation is significant at the 10%, 5%, and 1% level, respectively.
