# Supplementary material for: Smart city and earnings management: Evidence from China
Source: PLoS One. 2024 Apr 2;19(4):e0301025. doi: 10.1371/journal.pone.0301025 (PMC10986951; doi:10.1371/journal.pone.0301025)
Supplement: S2 Table — (DOCX) [file pone.0301025.s002.docx]

**Table S2:** Regression results of heterogeneity analysis.

| **Variable** | **(1) AEM–Smart City^b^** | | **(2) AEM–Smart City** |
| --- | --- | --- | --- |
| (a) Heterogeneity in regulatory intensity | | | |
|  | Higher regulatory intensity | Lower regulatory intensity | |
| Smart City×post | -0.003 | -0.008***^a^ | |
|  | (0.003)^c^ | (0.003) | |
| Observations | 10829 | 10305 | |
| R^2^ | 0.148 | 0.167 | |
| (b) Heterogeneity in marketization level | | | |
|  | Higher marketization level | Lower marketization level | |
| Smart City×post | -0.009** | -0.003 | |
|  | (0.003) | (0.002) | |
| Observations | 10437 | 10697 | |
| R2 | 0.156 | 0.162 | |
| (c) Heterogeneity in market concentration | | | |
|  | Lower market concentration | Higher market concentration | |
| Smart City×post | -0.007^**^ | -0.005 | |
|  | (0.003) | (0.004) | |
| Observations | 10602 | 10516 | |
| R2 | 0.162 | 0.161 | |
| (d) Heterogeneity across industries | | | |
|  | Manufacturing | Information | |
| Smart City∙post | -0.006^**^ | -0.056^***^ | |
|  | (0.003) | (0.009) | |
| Observations | 14087 | 1300 | |
| R^2^ | 0.131 | 0.275 | |
|  | Energy | Real estate & Construction | |
| Smart City×post | -0.006 | -0.060^***^ | |
|  | (0.009) | (0.017) | |
| Observations | 1174 | 1275 | |
| R^2^ | 0.350 | 0.315 | |
|  | Wholesale & Retail |  | |
| Smart City×post | 0.013 |  | |
|  | (0.009) |  | |
| Observations | 1149 |  | |
| R^2^ | 0.326 |  | |

^a^** and *** denote significance levels at the 5% and 1%, respectively.

^b^We control for time-fixed effects, industry-fixed effects, and city-fixed effects in all regressions.

^c^These regression models incorporate city-level clustered robust standard errors.
